# Seasonal variability of lesions distribution in acute ischemic stroke: A retrospective study

**DOI:** 10.1038/s41598-024-62631-w

**Published:** 2024-05-23

**Authors:** Xiao Sun, Xiaoshuang Xia, Juanjuan Xue, Yumeng Gu, Zhuangzhuang Chen, Peilin Liu, Fuyin Wang, Xiao Zhou, Jiaming Liu, Lin Wang, Xin Li

**Affiliations:** 1 Department of Neurology, The Second Hospital of Tianjin Medical University, Tianjin, China; 2 Department of Geriatrics, The Second Hospital of Tianjin Medical University, Tianjin, China; 3 Tianjin Center for Health and Meteorology Multidisciplinary Innovation, Tianjin, China

**Keywords:** Acute ischemic stroke, Meteorological risk warning, Lesions distribution, Seasonal variability, Stroke subtypes, Climate sciences, Diseases, Medical research, Neurology, Risk factors

## Abstract

Seasonal variability could have an impact on the incidence and outcome of stroke. However, little is known about the correlation between seasonal variability and location of acute cerebral infarction. This study aimed to explore the relationship between onset season and the lesions distribution of acute ischemic stroke (AIS). We retrospectively analysis data from 1488 AIS patients admitted to the Second Hospital of Tianjin Medical University from 2018 to 2022. All subjects completed head magnetic resonance imaging examination (MRI) and were divided into four groups according to the onset seasons. The lesions distribution of AIS was evaluated for anterior/posterior/double circulation infarction (DCI), unilateral/bilateral infarctions, and single/multiple cerebral infarctions based on MRI. Logistic regression models were employed to assess the association of season with lesions distribution of AIS. Subgroup analysis was performed in different stroke subtypes. Of 1488 patients, 387 (26.0%) AIS occurred in spring, 425 (28.6%) in summer, 331 (22.2%) in autumn and 345 (23.2%) in winter. Multivariate logistic regression demonstrated that the winter group had 2.15 times (95% CI:1.44–3.21) risk of multiple infarctions, 2.69 times (95% CI:1.80–4.02) of bilateral infarctions and 1.54 times (95% CI:1.05–2.26) of DCI compared with summer group, respectively. Subgroup analysis showed an increased risk of multiple (*p* < 0.01) or bilateral infarctions (*p* < 0.01) in small-artery occlusion (SAO) subtype, and higher risk of bilateral infarctions (*p* < 0.01) or DCI (*p* < 0.05) in large artery atherosclerosis (LAA) subtype during winter. No significant associations of season with lesions distribution in cardioembolism subtype. Our study highlighted a prominent seasonal variability in the lesions distribution of AIS, particularly in LAA and SAO subtypes. The findings could help to formulating meteorological risk warning strategies for different subtypes.

## Introduction

As a leading global health concern, stroke is a predominant cause of disability and mortality^1^. Ischemic stroke accounts for approximately 85% of all stroke cases, imposing a heavy burden on both society and families^2^. The pathogenesis of ischemic stroke involves the complicated process, including thrombosis, inadequate cerebral blood flow, and subsequent hypoxic-ischemic injury to brain cells^3^. Beyond traditional vascular risk factors, extreme temperatures and seasonal variability have been confirmed as potential risk factors affecting stroke occurrence and outcome^4,5^.

The Global Burden of Diseases, Injuries, and Risk Factors Study (GBD) was first to determine the impact of non-optimal temperature on stroke in 2019^1^. Evidence have highlighted that stroke is particularly influenced by seasonal variability, notably with a predominance of peaks during winter^6,7^. Additionally, the season onset has been linked with severity and outcomes of ischemic stroke, and strokes occurring in winter are often more severe and have poorer outcomes compared to those in summer^8,9^.

However, existing studies largely focused on macro-level stroke incidence, mortality and prognostic assessments, with limited exploration into the effects on anatomical distribution of infarct lesions and the detailed implications of seasonal variability. It is well-established that the different territories involvement of ischemic stroke is linked with distinct clinical manifestations and functional outcome^10,11^. For instance, posterior circulation strokes are more likely to have a poor outcome compared to anterior circulation strokes^12^. Moreover, multiple infarctions are linked to more severe clinical manifestations, functional outcomes and the increased risk of complications, such as deep venous thrombosis and myocardial infarction^13^.

Therefore, this study aimed to explore the potential association of onset season with the lesion distribution of acute cerebral infarction, in order to provide deeper insights into the effects of seasonal variability on acute ischemic stroke (AIS) and help to establishing meteorological risk warning strategies for AIS.

## Methods

### Study population

**Figure 1:**
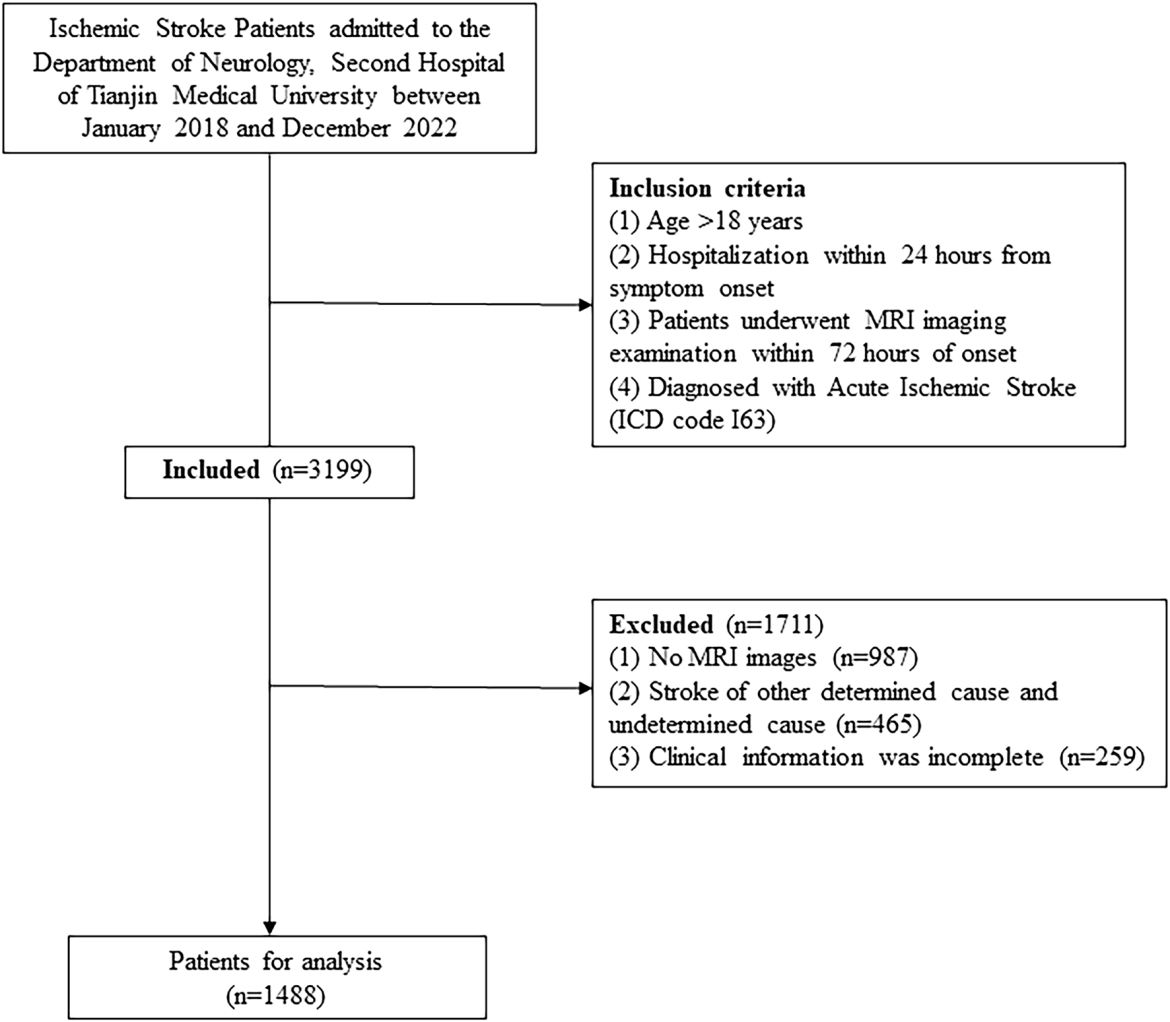
Flowchart of study enrollment.

The study population was patients admitted to the Department of Neurology, Second Hospital of Tianjin Medical University due to AIS (ICD code I63) between January 2018 and December 2022 (n = 3199). The inclusion criteria in this study were as follows: (1) Age > 18 years; (2) Hospitalization within 24 h from symptom onset; (3) Patients underwent MRI imaging examination within 72 h of onset; (4) Diagnosed with Acute Ischemic Stroke (ICD code I63). The exclusion criteria were as follows: (1) No MRI images (n = 987); (2) Stroke of other determined cause and undetermined cause (n = 465); (3) Clinical information was incomplete (n = 259). The comparison of demographic variables between the enrolled and excluded patients are shown in the Supplement Data-Table S1. Finally, a total of 1488 patients were analyzed in our study. The study flow chart is shown in Fig. 1.

### Data collection

We collected demographic and clinical data including age, sex, medical history (hypertension, diabetes, stroke, hyperlipidaemia, coronary heart disease (CHD), atrial fibrillation (AF), smoking status and alcohol consumption), onset time, National Institutes of Health Stroke Scale (NIHSS), systolic blood pressure (SBP) and diastolic blood pressure (DBP) and certain laboratory findings which were evaluated within 24 h of admission. Data collection was obtained by reviewing the medical records of the stroke admission.

According to the Trial of ORG 10172 in Acute Stroke Treatment (TOAST) classification system^14^, AIS was classified into the following 5 categories: (1) large artery atherosclerosis (LAA); (2) small-artery occlusion (SAO); (3) cardioembolism (CE); (4) stroke of other determined cause; and (5) stroke of undetermined cause. The diagnosis of stroke subtypes was determined by experienced clinical doctors based on clinical features, such as brain imaging (CT/MRI), vascular imaging (MRA/Transcranial Doppler/carotid artery ultrasound), echocardiography, and laboratory assessments. In this study, stroke of other determined cause and undetermined cause were excluded. The NIHSS was used to assess the neurological function, with stroke severity classified into four levels: minor (NIHSS ≤ 5), mild (NIHSS 6–10), moderate (NIHSS 11–15), and severe (NIHSS ≥ 16)^15^. Patients were divided into 4 groups according to the season of stroke onset: spring (March–May), summer (June–August), autumn (September–November), and winter (December-February).

Ethical considerations were in accordance with the Declaration of Helsinki, and the study was approved by the Institutional Review Board of the Second Hospital of Tianjin Medical University. Informed consent was waived due to the retrospective nature of the study.

### Evaluation of lesion distribution on MRI

In this study, all patients underwent MRI imaging examination within 72 h of onset. The diagnosis of AIS was based on established criteria^16^. The lesions of acute cerebral infarctions were categorized as anterior circulation infarction (ACI), posterior circulation infarction (PCI), or anterior and posterior double circulation infarction (DCI) based on involved vascular territories. According to established criteria^17–20^, the vascular territories in the anterior circulation included the anterior cerebral artery (ACA), middle cerebral artery (MCA), anterior choroidal artery, and border zone. In the posterior circulation, the vascular territories are the superior posterior cerebral artery (PCA), basilar artery (BA), superior cerebellar artery (SCA), anterior inferior cerebellar artery (AICA), and the posterior inferior cerebellar artery (PICA). Acute cerebral infarctions were also classified as unilateral or bilateral based on the location of the lesion. The unilateral (left/right) cerebral infarction is defined as new infarction only on one side of the midline structure of the brain, and the bilateral cerebral infarction is defined as the presence of intracranial infarction on both sides of the midline structure^18,19^. Single cerebral infarction refers to a single continuous lesion within the same blood supply range. Multiple cerebral infarctions are defined as ≥ 2 lesions located in one or more major arterial territories on DWI that was separated at the scanning level^21^.

### Statistical analysis

All analyses were conducted using IBM Statistical Package for the Social Sciences Statistics V27.0 for Windows (IBM Corp., released 2020, Armonk, NY, USA). Continuous variables were described with mean ± standard deviation (SD) or median and interquartile range (IQR). Categorical variables were presented as numbers and percentages. Continuous variables were compared using t-tests or analysis of variance (ANOVA) for normally distributed data and Mann–Whitney U test or Kruskal-Walli’s test for non-normally distributed data. Categorical variables were compared using the Chi-square test or Fisher’s exact test as appropriate. The Bonferroni adjustment was performed to assess the statistical significance of the intergroup differences. Logistic regression was applied for calculating the associations between season and lesions distribution of acute cerebral infarction. The results were reported as odds ratio (OR) values and their 95% confidence intervals (CI). Subgroup analysis was performed in different stroke subtypes. All statistical tests were two-tailed, and *P* < 0.05, indicated statistical significance.

## Results

### Demographic and clinical characteristics of study populations

The demographic and clinical characteristics of the study populations are shown in Table 1. Of the 1448 AIS patients enrolled in the study, the mean age was 70.7 ± 11.6 years old and 913 (61.4%) were male. According to the onset season, 387 (26.0%) AIS occurred in spring, 425 (28.6%) in summer, 331 (22.2%) in autumn and 345 (23.2%) in winter. In all patients, 604 (40.6%) patients were classified as LAA subtype, 308 (20.7%) as CE and 576 (38.7%) as SAO subtype. The incidence of LAA stroke was higher in winter, and CE or SAO stroke was higher in summer. Regarding the medical history, CHD (*p* < 0.001) and hyperlipemia (*p* < 0.001) showed significant seasonal differences. Clinical characteristics on admission showed a trend towards higher NIHSS in spring and winter as compared with summer. The severity showed significant seasonal variability (*p* < 0.001). Results of laboratory tests on admission showed that total cholesterol (*p* = 0.021) and fibrinogen (*p* = 0.018) level were significantly higher during spring and winter. No significant seasonal differences were registered for SBP and DBP on admission. Demographic and clinical characteristics of patients with different stroke subtypes are shown in the Supplement Data-Table S2.

**Table 1:** Demographic and clinical characteristics of study populations.

Variables	All (n = 1488)	Spring (n = 387)	Summer (n = 425)	Autumn (n = 331)	Winter (n = 345)	Overall p
Age (years)	70.7(11.6)	70.9 (11.5)	69.9 (11.3)	71.98 (11.6)	70.4 (11.9)	0.163
Male (%)	913 (61.4)	233 (25.5)	262 (28.7)	210 (23.0)	208 (22.8)	0.80
Hypertension (%)	1201 (80.7)	318 (26.5)	349 (29.1)	261 (21.7)	273 (22.7)	0.501
Diabetes, (%)	585 (39.3)	157 (26.8)	176 (30.1)	116 (19.8)	136 (23.2)	0.312
Stroke (%)	409 (27.5)	108 (26.4)	122 (29.8)	77 (18.8)	102 (24.9)	0.255
CHD (%)	290 (19.5)	69 (23.8)	68 (23.4)	93 (32.1)^**a**^	60 (20.7)	< 0.001
AF (%)	209 (14.0)	54 (25.8)	57 (27.3)	55 (26.3)	43 (20.6)	0.445
Hyperlipemia (%)	140 (9.4)	18 (12.9)^**a**^	52 (37.1)	45 (31.2)^**a**^	25 (17.9)^**a**^	< 0.001
Drinking (%)	347 (23.3)	81 (23.3)	92 (26.5)	90 (25.9)	84 (24.2)	0.181
Smoking (%)	558 (37.5)	142 (25.4)	156 (28.0)	127 (22.8)	133 (23.8)	0.921
SDP (mmHg)	154 (22)	155 (23)	152 (22)	154 (23)	154 (21)	0.115
DBP (mmHg)	87 (14)	88 (14)	87 (14)	86 (14)	88 (15)	0.167
NIHSS	3 (1, 7)	3 (1, 8)	2(1, 5)	2(1, 6)	3(1, 8)	0.05
Severity level						< 0.001
Minor (%)	1020 (68.5)	256 (25.1)^**a**^	323 (31.7)	232 (22.7)^**a**^	209 (20.5)^**a**^	
Mild (%)	271 (18.2)	73 (26.9)^**a**^	60 (22.1)	53 (19.6)	85 (31.4)^**a**^	
Moderate (%)	94 (6.3)	31 (33.0)^**a**^	14 (14.9)	22 (23.4)^**a**^	27 (28.7)^**a**^	
Severe (%)	103 (6.9)	27 (26.2)	28 (27.2)	24 (23.3)	24 (23.3)	
Stroke subtypes						0.015
LAA (%)	604 (40.6)	156 (25.8)^**a**^	149 (24.7)	140 (23.2)^**a**^	159 (26.3)^**a**^	
CE (%)	308 (20.7)	71 (23.1)^**a**^	100 (32.5)	78 (25.3)^**a**^	59 (19.2)^**a**^	
SAO (%)	576 (38.7)	160 (27.8)^**a**^	176 (30.6)	113 (19.6)^**a**^	127 (22.0)^**a**^	
FBG (mmol/L)	7.19 (3.2)	7.11 (2.9)	7.20 (3.2)	6.95 (3.3)	7.54 (3.8)	0.116
TG (mmol/L)	1.58 (1.0)	1.64 (1.2)	1.54 (0.8)	1.50 (0.8)	1.64 (1.2)	0.103
TC (mmol/L)	4.79 (1.2)	4.92 (1.2)^**a**^	4.67 (1.2)	4.74 (1.2)	4.83 (1.2)^**a**^	0.021
LDL (mmol/L)	3.07 (1.0)	3.16 (0.9)	3.02 (0.9)	3.03 (1.0)	3.10 (1.0)	0.140
HDL (mmol/L)	1.13 (0.3)	1.14 (0.3)	1.10 (0.3)	1.14 (0.3)	1.14 (0.3)	0.100
PLT (× 10^9^/L)	217 (176, 246)	218 (177, 243)	214 (175, 248)	214 (173, 238)	217 (178, 257)	0.174
Fibrinogen (g/L)	3.4 (2.7, 3.7)	3.4 (2.7, 3.9)^**a**^	3.2 (2.6, 3.5)	3.4 (2.7, 3.7)	3.4 (2.7, 3.7)^**a**^	0.018

CHD coronary heart disease, AF atrial fibrillation, SBP systolic blood pressure, DBP diastolic blood pressure, LAA large-artery atherosclerosis, CE cardioembolism, SAO small-artery occlusion, FBG fasting blood glucose, TG triglyceride, TC total cholesterol, LDL low-density lipoprotein cholesterol, HDL high-density lipoprotein cholesterol, PLT blood platelet.

Overall p-value is for the test of difference among the 4 season groups.

^a^Significantly different from summer group (the Bonferroni correction was applied).

### Lesion distribution of AIS in different seasons

The results of lesion distribution of acute ischemic stroke in different seasons are shown in Table 2. Among all the cases, ACI occurred in 678 (45.6%) patients, PCI occurred in 405 (27.2%), and DCI in 405 (27.2%), respectively. There are 719 (48.3%) patients diagnosed with single infarction, and 769 (51.7%) patients diagnosed with multiple infarctions. 1189 (79.9%) patients have unilateral lesions, and 299 (20.1%) have bilateral lesions. There are significant seasonal differences in the distribution of lesions on DWI. The incidence of ACI was lower in winter than that in summer (*p* < 0.05). The incidence of PCI or DCI showed a trend towards higher in winter, but no significant differences. Moreover, multiple infarctions or bilateral infarctions was more likely to occur in winter compared with summer (all *p* < 0.05).

**Table 2:** Lesion distribution of acute ischemic stroke in different seasons

	All (n = 1488)	Spring (n = 387)	Summer (n = 425)	Autumn (n = 331)	Winter (n = 345)	Overall p
Territory						0.004
ACI (%)	678 (45.6)	193 (28.5)	207 (30.5)	155 (22.9)	123 (18.1)*	
PCI (%)	405 (27.2)	93 (23.0)	111 (27.4)	90 (22.2)	111 (27.4)	
DCI (%)	405 (27.2)	101 (24.9)	107 (26.4)	86 (21.2)	111 (27.4)	
Number of lesions						0.006
Single (%)	719 (48.3)	188 (26.1)	226 (32.4)	165 (22.9)	140 (19.5)*	
Multiple (%)	769 (51.7)	199 (25.9)	199 (25.9)	166 (21.6)	205 (26.7)*	
Location						0.003
Unilateral (%)	1189 (79.9)	322 (27.1)	351 (29.5)	263 (22.1)	253 (21.3)*	
Bilateral (%)	299 (20.1)	65 (21.7)	74 (24.7)	68 (22.7)	92 (30.8)*	

ACI, anterior circulation infarction; PCI, posterior circulation infarction; DCI, double-circulation infarction.

Overall p-value is for the test of difference among the 4 season groups.

*Significantly different from summer group (the Bonferroni correction was applied).

### Lesion distribution of AIS in various seasons with different stroke subtypes

Subgroup analysis found significant seasonal differences in the distribution of lesions of LAA and SAO stroke subtypes, but not in CE subtype (Table 3). In LAA stroke subtype, there are significant seasonal differences in the number of lesions (*p* = 0.025) and location of infarctions (unilateral/ bilateral lesions, *p* = 0.038). Compared with summer, multiple infarctions and bilateral infarctions are more common in spring and winter (all *p* < 0.05). No significant seasonal variations in the territory of infarctions. Among ASO stroke patients, DCI only have 2 (0.4%) cases, and more than 80% of patients are single (89.2%) or unilateral (88.4%) infarctions. There are significant seasonal differences in the distribution of lesions in SAO stroke subtype. The incidence of ACI can be seen lower but PCI higher in winter. Furthermore, the incidence of multiple infarctions and bilateral infarctions are higher in winter (all *p* < 0.05).

**Table 3:** Lesion distribution of acute ischemic stroke in various seasons by stroke subtypes.

LAA	All (n = 605)	Spring (n = 156)	Summer (n = 149)	Autumn (n = 140)	Winter (n = 159)	Overall p
Territory						0.095
ACI (%)	263 (43.6)	67 (25.5)	74 (28.1)	63 (24.0)	59 (22.4)	
PCI (%)	87 (14.4)	18 (20.7)	20 (23.0)	27 (31.0)	22 (25.3)	
DCI (%)	254 (42.0)	71 (28.0)	55 (21.7)	50 (19.7)	78 (30.7)	
Number of lesions						0.025
Single (%)	145 (24.0)	27 (18.6)	33 (22.8)	45 (31.0)	40 (27.6)	
Multiple (%)	459 (76.0)	129 (28.1) *	116 (25.3)	95 (20.7)	119 (25.9) *	
Location						0.038
Unilateral (%)	501 (82.8)	126 (25.1)	135 (26.9)	111 (22.2)	129 (25.7)	
Bilateral (%)	103 (17.2)	30 (29.1) *	14 (13.6)	29 (28.2) *	30 (29.1) *	

CE	All (n = 308)	Spring (n = 71)	Summer (n = 100)	Autumn (n = 78)	Winter (n = 59)	Overall p
Territory						0.301
ACI (%)	117 (38.0)	33 (28.2)	33 (28.2)	34 (29.1)	17 (14.5)	
PCI (%)	42 (13.6)	8 (19.0)	17 (40.5)	8 (19.0)	22 (21.4)	
DCI (%)	149 (48.4)	30 (20.1)	50 (33.6)	36 (24.2)	33 (22.1)	
Number of Lesions						0.577
Single (%)	60 (19.5)	15 (25.0)	23 (38.3)	13 (21.7)	9 (15.0)	
Multiple (%)	248 (80.5)	56 (22.6)	77 (31.0)	65 (26.2)	50 (20.2)	
Distribution						0.172
Unilateral (%)	179 (58.1)	47 (26.3)	50 (27.9)	48 (26.8)	34 (19.0)	
Bilateral (%)	129 (41.9)	24 (18.6)	50 (38.8)	30 (23.3)	25 (19.4)	

SAO	All (n = 576)	Spring (n = 156)	Summer (n = 149)	Autumn (n = 140)	Winter (n = 159)	Overall p
Territory						0.002
ACI (%)	298 (51.7)	93 (31.2)	100 (33.6)	58 (19.5)	47 (15.8)*	
PCI (%)	276 (47.9)	67 (24.3)	74 (26.8)	55 (19.9)	80 (29.0)*	
DCI (%)	2 (0.4)	0 (0)	2 (100)	0 (0)	0 (0)	
Number of Lesions						< 0.001
Single (%)	514 (89.2)	146 (28.4)	170 (33.1)	107 (20.8)	91 (17.7)*	
Multiple (%)	62 (10.8)	14 (22.6)	6 (9.7)	6 (9.7)	36 (58.1)*	
Distribution						< 0.001
Unilateral (%)	509 (88.4)	149 (29.3)	166 (32.6)	104 (20.4)	90 (17.7)*	
Bilateral (%)	67 (11.6)	11 (16.4)	10 (14.9)	9 (13.4)	37 (55.2)*	

*ACI* anterior circulation infarctions, *PCI* posterior circulation infarctions, *DCI* double circulation infarctions.

*Significantly different from summer group (The Bonferroni correction was applied).

### Logistic regression analysis for season and lesion distribution of AIS

After adjusting for age, sex, stroke and other confounding factors, multivariate logistic regression demonstrated that the winter group had 2.15 times (95% CI:1.44–3.21) risk of multiple infarctions, 1.54 times (95% CI:1.05–2.26) of bilateral infarctions and 2.69 times (95% CI:1.80–4.02) of DCI compared with summer group, respectively (Table 4).

**Table 4:** Logistic regression analysis of the association between season and lesion distribution of acute ischemic stroke.

	Multiple infarctions		Bilateral infarctions		DCI	
	Unadjusted OR 95% CI	Adjusted OR 95% CI	Unadjusted OR 95% CI	Adjusted OR 95% CI	Unadjusted OR 95% CI	Adjusted OR 95% CI
Spring	1.20 (0.91, 1.58)	1.51 (1.03, 2.21)	1.05 (0.77, 1.44)	1.23 (0.84, 1.80)	0.96 (0.66, 1.38)	1.38 (0.91, 2.11)
Summer	Reference	Reference	Reference	Reference	Reference	Reference
Autumn	1.14 (0.86, 1.52)	1.01 (0.69, 1.49)	1.04 (0.75, 1.45)	0.98 (0.66, 1.44)	1.23 (0.85, 1.77)	1.64 (1.07, 2.51)^*^
Winter	1.66 (1.25, 2.22)^**^	2.15 (1.44, 3.21)^**^	1.41 (1.03, 1.93)^*^	1.54 (1.05, 2.26)^*^	1.73 (1.22, 2.44)^**^	2.69 (1.80, 4.02)^**^

Adjusted for sex, age, stroke, hypertension, diabetes, CHD, atrial fibrillation, hyperlipemia, smoking, drinking, TOAST classification.

*DCI* double circulation infarctions, *CI* confidence interval, *OR* odds ratio.

*p < 0.05, **p < 0.01.

**Figure 2:**
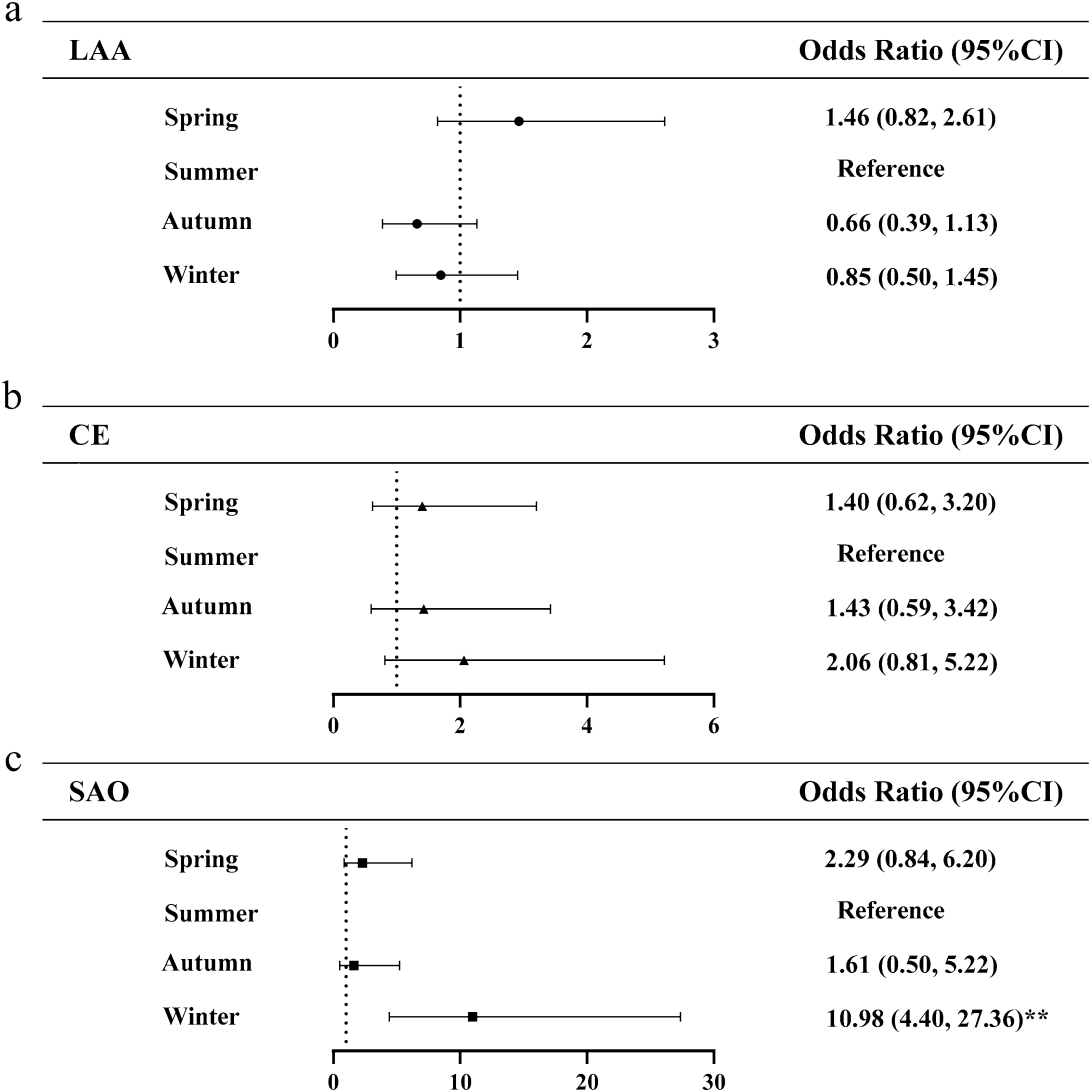
Multivariate logistic regression analysis of the association between season and multiple cerebral infarctions. *LAA* large-artery atherosclerosis, *CE* cardioembolism, *SAO* small-artery occlusion. The ORs were calculated using logistic regression model after adjusting sex, age, stroke, hypertension, diabetes, CHD, atrial fibrillation, hyperlipemia, smoking, drinking. ^*^p < 0.05, ^**^p < 0.01.

**Figure 3:**
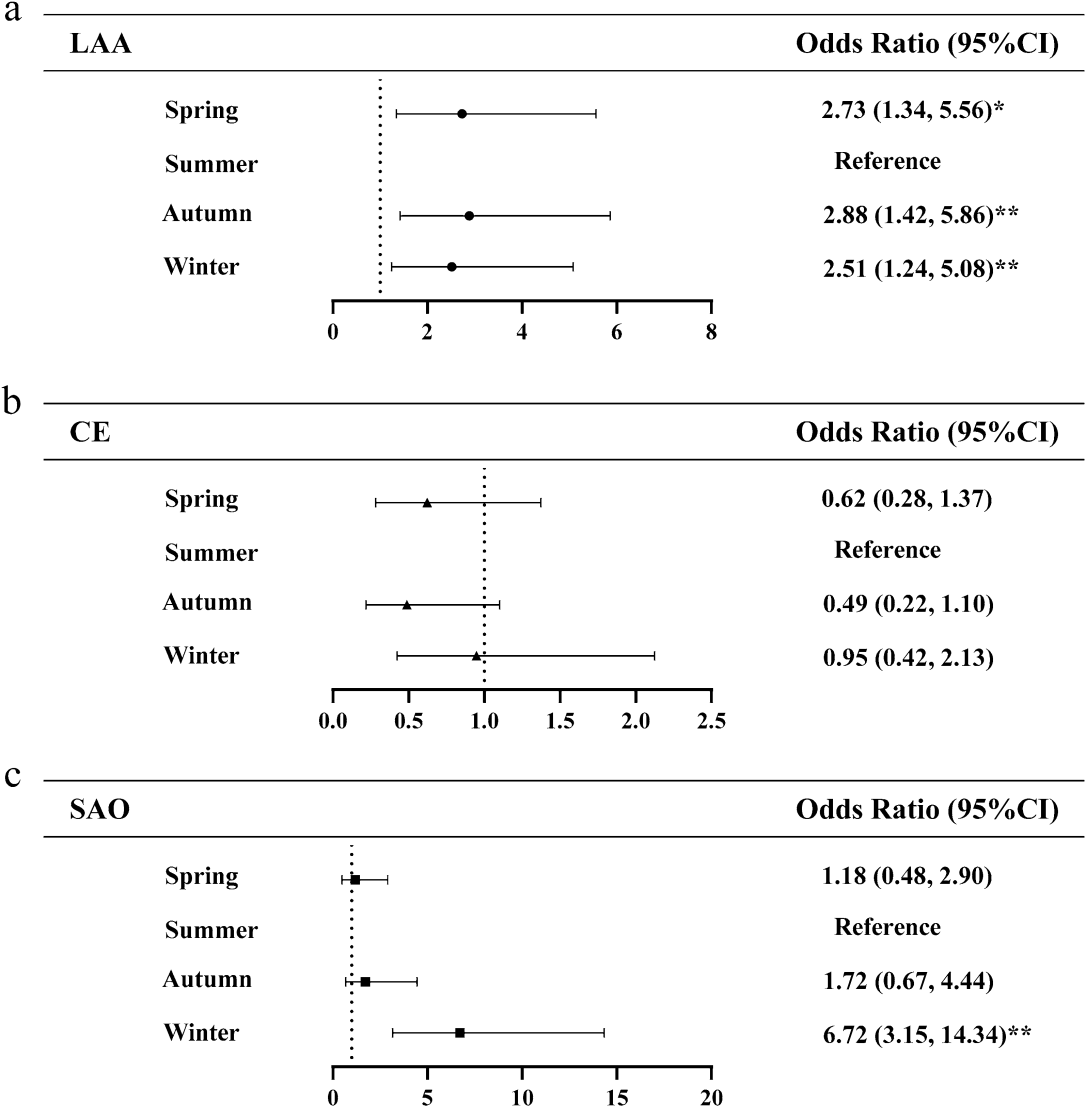
Multivariate logistic regression analysis of the association of season with bilateral cerebral infarctions. Abbreviations: *LAA* large-artery atherosclerosis, *CE* cardioembolism, *SAO* small-artery occlusion. The ORs were calculated using logistic regression model after adjusting sex, age, stroke, hypertension, diabetes, CHD, atrial fibrillation, hyperlipemia, smoking, drinking. ^*^p < 0.05, ^**^p < 0.01.

**Figure 4:**
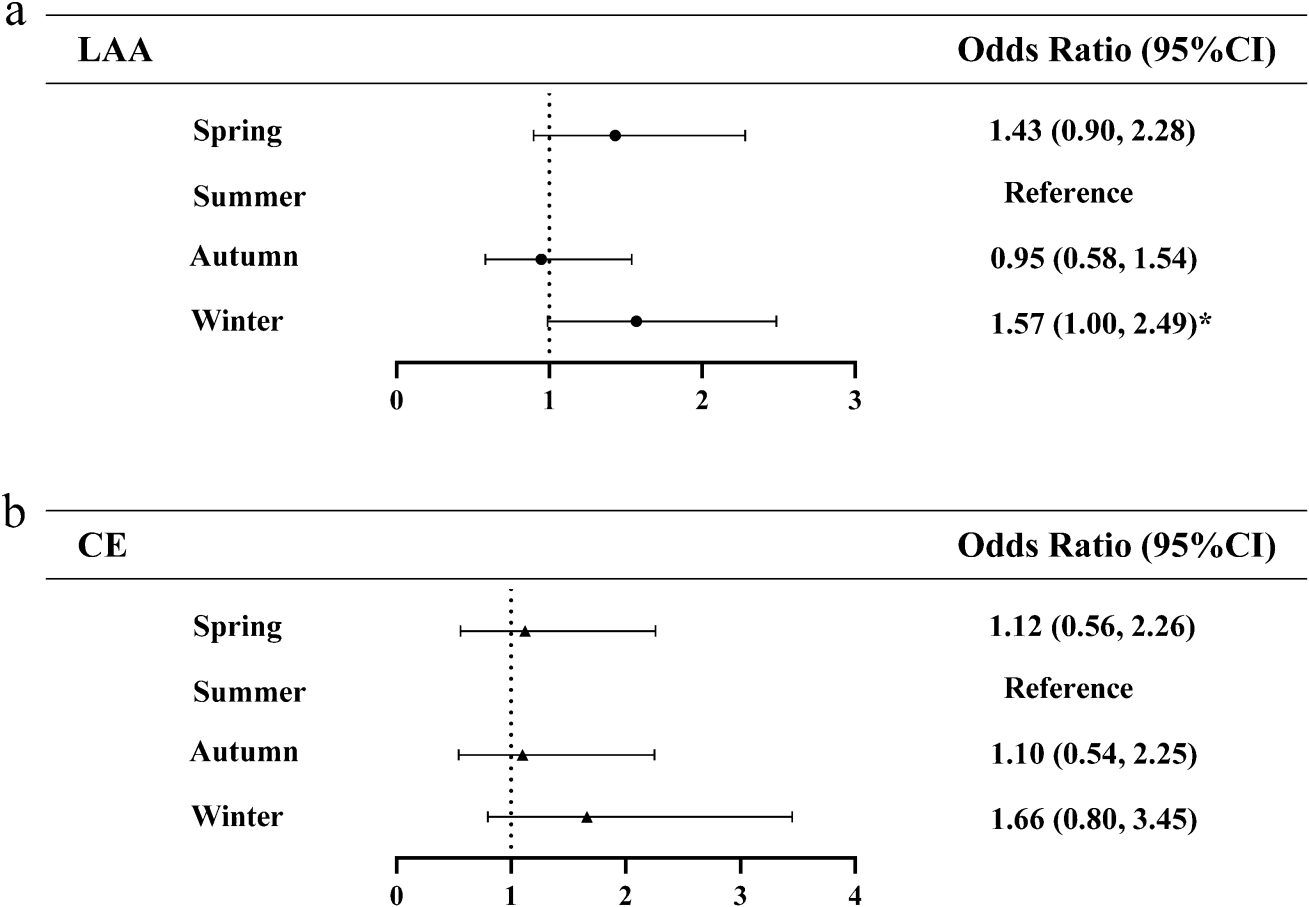
Multivariate logistic regression analysis of the association of season with double circulation cerebral infarctions Abbreviations: LAA, large-artery atherosclerosis; CE, cardioembolism; DCI, double circulation cerebral infarcts. The ORs were calculated using logistic regression model after adjusting sex, age, stroke, hypertension, diabetes, CHD, atrial fibrillation, hyperlipemia, smoking, drinking. ^*^p < 0.05, ^**^p < 0.01.

Subgroup analysis showed that the association of season with infarct distribution differed among 3 stroke subtypes. As shown in Fig. 2, multivariate logistic regression revealed that the risk of multiple infarctions in SAO stroke subtype increased to 10.98 times in winter (95% CI 4.40–27.36, *p* < 0.01) compared with summer group. No significant associations of season with multiple infarctions in LAA and CE stroke subtypes. Figure 3 shows the association of season with bilateral infarctions in 3 stroke subtypes. Among LAA stroke subtype, compared with summer, the risk of bilateral cerebral infarctions increased to 2.73 times in spring (95% CI 1.34–5.56,* p* < 0.05), 2.88 times in autumn (95% CI 1.42–5.86, *p* < 0.01) and 2.51 times in winter (95% CI 1.24–5.08, p < 0.01), respectively. And multivariate logistic regression indicated that winter (95% CI 3.15–14.34, *p* < 0.01) is significantly associated with bilateral cerebral infarctions in SAO stroke patients. Significant associations of season with bilateral infarctions were not revealed in CE stroke subtypes. Compared with summer, risk of DCI in LAA stroke subtype increased to 1.57 times in winter (95%CI 1.00–2.49, *p* < 0.05). No significant associations of season with DCI in CE stroke subtype. The SAO stroke subtype was not analyzed owning to the small number of DCI (Fig. 4).

## Discussion

In this study, our findings have demonstrated significant seasonal variability in distribution of infarctions lesions in AIS patients, particularly in LAA and SAO stroke subtypes. Specifically, there was an increased risk of multiple or bilateral infarcts in SAO stroke patients and a higher risk of anterior and posterior DCI in LAA stroke patients during winter. However, it’s intriguing to note that in cardioembolic stroke, the distribution of infarction lesions was not shown significant seasonal variation.

Previous research has established a correlation between seasonal factors and an increased incidence and mortality of stroke, particularly during colder winter, attributing this trend to lower ambient temperatures or greater amplitude of temperatures^5,22^. Lower temperatures may lead to elevating blood pressure, activating platelets and increasing blood viscosity, which potentially impact vascular function and increase the risk of stroke^23^. Furthermore, the season of onset has been associated with the severity and outcomes of ischemic stroke, with cases occurring in winter generally presenting more severe symptoms and poorer outcomes compared to those in summer^8,9^. More importantly, the location of cerebral infarction plays a crucial role in determining clinical presentation, comorbidities and outcome^24^. Studies showed that complications were more frequent in patients with multiple cerebral infarctions, which can adversely affect short-term clinical and functional outcome^13^. To the best of our knowledge, this is the first study to investigate the seasonal variability in distribution of infarctions lesions in AIS patients. The findings may contribute to shedding light on the influence of season on stroke occurrence and prognosis.

The present study revealed that patients with LAA stroke had higher risk of bilateral or DCI infarctions during winter compared to summer. For LAA stroke patients, bilateral infarctions, especially those occurring in multiple circulations often pointed to a more proximal source of embolism, such as aortic arch arteriosclerosis^25,26^. The potential reasons for increased risk of bilateral or DCI infarctions during winter are as follows. Firstly, the cold-mediated plaque instability may be one of the important reasons. Evidence suggests that cold exposure via uncoupling protein 1 (UCP1)-dependent activates lipolysis, leading to elevated levels of blood cholesterol and low density lipoprotein (LDL) cholesterol, especially very LDL (VLDL) and small LDL remnants, which promotes atherosclerotic plaque growth and instability^27^. Moreover, cold can enhance the instability of atherosclerotic plaques by activating endoplasmic reticulum stress, promoting cellular apoptosis, and mediating inflammation response leading to the rupture of vulnerable plaques^28,29^. Above all, the lower ambient temperatures during winter may cause aortic plaques rupture into several fragments, which could embolize different arterial segments of the cerebral circulation^30^. Secondly, patients with LAA stroke generally possess a higher burden of vascular risk factors, and the seasonal and temperature variability of these risk factors (e.g., blood pressure, blood glucose) may further contribute to the progression and rupture of atherosclerotic plaques^31,32^. Thirdly, cold spells were found to be significantly correlated with increased hematocrit and fibrinogen level, which may increase the tendency for blood clots to form during cold winter^33,34^. Certainly, the specific mechanism needs to be further confirmed by more in-depth basic experiments.

Our study indicated the increased risk of multifocal or bilateral infarctions in SAO stroke during winter. The leading cause of small-artery disease include arteriolosclerosis, microatheroma and lipohyalinosis^35^. Chronic hypertension is considered as the primary cause of these pathological events, but other diseases, such as aging, atherosclerosis, and diabetes can lead to brain microcirculation impairment ^36^. The exact mechanism leading to multiple ischemic lesions is currently unknown. But systemic factors like blood pressure regulation, hemorheological factors, endothelial dysfunction, and potentially stress might play a role ^37^. Studies proved that blood pressure exhibited significant seasonal variability with a winter peak, especially in the elderly population^38,39^, which could be attributed to lower ambient temperature. Additionally, the adverse effects of decreased temperature on small vessels, potentially involving temperature-dependent vascular constriction, altered hemodynamics, or metabolic dysregulation within microcirculation^40,41^. Consequently, the lower temperature may cause diffuse damage to small vessels and increase the risk of multiple lesions in winter^37^. So, additional preventive measures for patients with small-vessel occlusion might be prudent during winter, such as maintaining ambient warmth, adequate body insulation, and potentially vascular-protective medical interventions.

In this study, there is no significant seasonal variability in the distribution of lesions in CE stroke patients. Studies confirmed that multiple infarctions and involves multiple vascular territories (combined anterior and posterior) mostly point to a cardiac origin of the stroke^42^, which is consistent with our findings. This infarction pattern may be related to the underlying mechanisms of cardioembolic strokes. Cardioembolic stroke is typically caused by embolism formation due to heart diseases which include arrhythmia, valvulopathy, and structural abnormalities (such as atrial septal defect and patent foramen ovale)^43^. AF, as a disorder of heart rhythm, is considered as the most common risk factor of cardioembolic stroke. Evidence suggests that declining ambient temperature could increase the risk of AF episode onset^44^, which may contribute to the increased incidence of cardioembolic stroke in cold season^45^. However, there is still a lack of sufficient evidence on whether the increased risk of AF mediated by low temperatures affects the seasonal distribution of infarctions lesion in cardioembolic strokes. More studies are needed to explore the association between this increased risk of AF and seasonal differences in lesion distribution in the future.

Our study has several strengths. The study firstly investigated the seasonal variability in distribution of infarcts lesions in different stroke subtypes. Furthermore, the lesions distribution of AIS was confirmed by brain imaging ensuring accurate diagnosis and classification. Nevertheless, some limitations of our study should be acknowledged. First, the sample is from a single center and has small size, which may not reflect the overall seasonal variation trend of AIS across different populations and climates around the world. Thus, caution is advised in generalizing our findings globally. Second, aortic arch atherosclerosis may be an overlooked cause of stroke because transesophageal echocardiography was not commonly performed for stroke patients in our clinical practice. This may lead to an underestimation of LAA stroke subtypes. Third, the exact location (intracranial vs extracranial) of atherosclerotic lesions was not systematically assessed in this study, which will be addressed in our future research. Additionally, the retrospective design of our study precludes the determination of causality, warranting prospective, multi-center studies to further validate the observed impacts of seasonal variability on lesion distribution in different stroke subtypes.

In conclusion, our study suggested a significant association between onset season and the distribution of infarcts lesions in AIS patients. During winter, there is a significantly increased risk of multiple or bilateral or dual circulation cerebral infarction during winter, particularly in LAA and SAO stroke subtypes. Our findings provide novel insights into the effects of seasonal variability on various stroke subtypes, which may conduce to establishing new preventive strategies and meteorological risk warning system for different stroke subtypes.

### Supplementary Information

Supplementary Tables.

## Data Availability

The data that support the findings of this study are available from the corresponding author upon reasonable request.

## References

[1] Global, regional, and national burden of stroke and its risk factors, 1990-2019: a systematic analysis for the Global Burden of Disease Study 2019. *The Lancet. Neurology* **20**, 795-820, 10.1016/s1474-4422(21)00252-0 (2021). 10.1016/S1474-4422(21)00252-0 PMC8443449 34487721

[2] Ding Q (2022). Global, regional, and national burden of ischemic stroke, 1990–2019. Neurology.

[3] Qin C (2022). Signaling pathways involved in ischemic stroke: Molecular mechanisms and therapeutic interventions. Signal Trans. Target. Ther..

[4] Yang J (2016). The burden of stroke mortality attributable to cold and hot ambient temperatures: Epidemiological evidence from China. Environ. Int..

[5] Chen R (2018). Association between ambient temperature and mortality risk and burden: Time series study in 272 main Chinese cities. BMJ.

[6] Alghamdi SAM (2021). Stroke seasonality and weather association in a Middle East country: A single tertiary center experience. Front. Neurol..

[7] Qi X (2020). Potential impacts of meteorological variables on acute ischemic stroke onset. Risk Manag. Healthcare Policy.

[8] Liu Y, Gong P, Wang M, Zhou J (2018). Seasonal variation of admission severity and outcomes in ischemic stroke—A consecutive hospital-based stroke registry. Chronobiol. Int..

[9] Xue J (2023). Seasonal variation in neurological severity and clinical outcomes in ischemic stroke patients—A 9-Year study of 5,238 patients. Circul. J..

[10] Novotny V (2021). Clinical manifestation of acute cerebral infarcts in multiple arterial territories. Brain Behav..

[11] Bamford J, Sandercock P, Dennis M, Burn J, Warlow C (1991). Classification and natural history of clinically identifiable subtypes of cerebral infarction. Lancet.

[12] Sommer P (2018). Is functional outcome different in posterior and anterior circulation stroke?. Stroke.

[13] Novotny V (2019). Short-term outcome and in-hospital complications after acute cerebral infarcts in multiple arterial territories. Stroke.

[14] Adams HP (1993). Classification of subtype of acute ischemic stroke. Definitions for use in a Multicenter clinical trial. TOAST. Trial of Org 10172 in Acute Stroke Treatment. Stroke.

[15] Koton S (2022). Association of ischemic stroke incidence, severity, and recurrence with dementia in the atherosclerosis risk in communities cohort study. JAMA Neurol..

[16] Singer MB (1998). Diffusion-weighted MRI in acute subcortical infarction. Stroke.

[17] Frid P (2020). Detailed phenotyping of posterior vs. anterior circulation ischemic stroke: A multi-center MRI study. J. Neurol..

[18] Tatu L, Moulin T, Bogousslavsky J, Duvernoy H (1996). Arterial territories of human brain: Brainstem and cerebellum. Neurology.

[19] Tatu L, Moulin T, Bogousslavsky J, Duvernoy H (1998). Arterial territories of the human brain: Cerebral hemispheres. Neurology.

[20] Caso V, Budak K, Georgiadis D, Schuknecht B, Baumgartner RW (2005). Clinical significance of detection of multiple acute brain infarcts on diffusion weighted magnetic resonance imaging. J. Neurol. Neurosurg. Psychiatry.

[21] Wong KS (2002). Mechanisms of acute cerebral infarctions in patients with middle cerebral artery stenosis: A diffusion-weighted imaging and Microemboli monitoring study. Ann. Neurol..

[22] Kurtz P, Bastos LS, Aguilar S, Hamacher S, Bozza FA (2021). Effect of seasonal and temperature variation on hospitalizations for stroke over a 10-year period in Brazil. Int. J. Stroke.

[23] Chen Z, Liu P, Xia X, Wang L, Li X (2022). The underlying mechanisms of cold exposure-induced ischemic stroke. Sci.Total Environ..

[24] Simaan N (2023). Characteristics of multiple acute concomitant cerebral infarcts involving different arterial territories. J. Clin. Med..

[25] Baird AE, Lövblad KO, Schlaug G, Edelman RR, Warach S (2000). Multiple acute stroke syndrome: Marker of embolic disease?. Neurology.

[26] Darby DG, Parsons MW, Barber PA, Davis SM (2000). Significance of acute multiple brain infarction on diffusion-weighted imaging. Stroke.

[27] Dong M (2013). Cold exposure promotes atherosclerotic plaque growth and instability via UCP1-dependent lipolysis. Cell. Metabol..

[28] Ma T (2013). Th17 cells and IL-17 are involved in the disruption of vulnerable plaques triggered by short-term combination stimulation in apolipoprotein E-knockout mice. Cell. Mol. Immunol..

[29] Dai MX (2014). The impact of intermittent and repetitive cold stress exposure on endoplasmic reticulum stress and instability of atherosclerotic plaques. Cell. Physiol. Biochem..

[30] Ntaios G (2019). Aortic arch atherosclerosis in patients with embolic stroke of undetermined source: An exploratory analysis of the NAVIGATE ESUS Trial. Stroke.

[31] Zheng S (2021). Effects of cold and hot temperature on metabolic indicators in adults from a prospective cohort study. Sci. Total Environ..

[32] Stergiou GS (2020). Seasonal variation in blood pressure: evidence, consensus and recommendations for clinical practice. Consensus statement by the European society of hypertension working group on blood pressure monitoring and cardiovascular variability. J. Hypertens..

[33] Cheng BJ (2023). Short-term effects of cold spells on hematocrit among adults in Nanjing, China: A distributed-lagged effect analysis. Sci.Total Environ..

[34] Neild PJ (1994). Cold-induced increases in erythrocyte count, plasma cholesterol and plasma fibrinogen of elderly people without a comparable rise in protein C or factor X. Clin. Sci..

[35] Hainsworth AH, Markus HS, Schneider JA (2024). Cerebral small vessel disease, hypertension, and vascular contributions to cognitive impairment and dementia. Hypertension.

[36] Rovira A, Grivé E, Rovira A, Alvarez-Sabin J (2005). Distribution territories and causative mechanisms of ischemic stroke. Eur. Radiol..

[37] Norrving B (2004). Are multiple acute small subcortical infarctions caused by embolic mechanisms?. J. Neurol. Neurosurg. Psychiatry.

[38] Cepeda M, Muka T, Ikram MA, Franco OH, Schoufour JD (2018). Seasonality of insulin resistance, glucose, and insulin among middle-aged and elderly population: The Rotterdam study. J. Clin. Endocrinol. Metabol..

[39] Modesti PA (2013). Season, temperature and blood pressure: A complex interaction. Eur. J. Int. Med..

[40] Zhang D (2021). Reactive oxygen species are essential for vasoconstriction upon cold exposure. Oxid. Med. Cell. Long..

[41] Cai J (2016). The cold effects on circulatory inflammation, thrombosis and vasoconstriction in type 2 diabetic patients. Sci. Total Environ..

[42] Depuydt S (2014). Significance of acute multiple infarcts in multiple cerebral circulations on initial diffusion weighted imaging in stroke patients. J. Neurol. Sci..

[43] Kamel H, Healey JS (2017). Cardioembolic stroke. Circul. Res.

[44] Zhu X (2023). Low ambient temperature increases the risk and burden of atrial fibrillation episodes: A nationwide case-crossover study in 322 Chinese cities. Sci. Total Environ..

[45] Chen PY, Chang WL, Hsiao CL, Lin SK (2024). Seasonal variations in stroke and a comparison of the predictors of unfavorable outcomes among patients with acute ischemic stroke and Cardioembolic stroke. Biomedicines.

