# Supplementary material for: Seasonal variability of lesions distribution in acute ischemic stroke: A retrospective study
Source: Sci Rep. 2024 May 23;14:11831. doi: 10.1038/s41598-024-62631-w (PMC11116500; doi:10.1038/s41598-024-62631-w)
Supplement: Supplementary file 1 — Supplementary Tables. [file 41598_2024_62631_MOESM1_ESM.docx]

| **Table S1.** Comparison of demographic characteristics between enrolled and excluded patients | | | | | | | |
| --- | --- | --- | --- | --- | --- | --- | --- |
| Variables | | | Total  (n = 3199) | Inclusion  (n = 1488) | Exclusion  (n = 1711) | p |  |
| Age, mean (SD) | | 71 (11) | 71(11) | 71 (11) | 0.102 |  |  |
| Sex, n (%) |  |  |  | 0.212 |  |  |  |
| Male | 1917 (60) | 913 (61) | 1004 (59) |  |  |  |  |
| Female | 1282 (40) | 575 (39) | 707 (41) |  |  |  |  |
| Season, n (%) |  |  |  | 0.01 |  |  |  |
| Spring | 803 (25) | 387 (26) | 416 (24) |  |  |  |  |
| Summer | 813 (25) | 425 (29) | 388 (23) |  |  |  |  |
| Autumn | 766 (24) | 331 (22) | 435 (25) |  |  |  |  |
| Winter | 817 (26) | 345 (23) | 472 (28) |  |  |  |  |
| Stroke, n (%) |  |  |  | 0.090 |  |  |  |
| no | 2272 (71) | 1079 (73) | 1193 (70) |  |  |  |  |
| yes | 927 (29) | 409 (27) | 518 (30) |  |  |  |  |
| Hypertension, n (%) |  |  |  | 0.191 |  |  |  |
| no | 650 (20) | 287 (19) | 363 (21) |  |  |  |  |
| yes | 2549 (80) | 1201 (81) | 1348 (79) |  |  |  |  |
| Diabetes, n (%) |  |  |  | 0.106 |  |  |  |
| no | 1976 (62) | 903 (61) | 1073 (63) |  |  |  |  |
| yes | 1223 (38) | 585 (39) | 638 (37) |  |  |  |  |
| CHD, n (%) |  |  |  | 0.094 |  |  |  |
| no | 2373 (74) | 1198 (81) | 1175 (70) |  |  |  |  |
| yes | 826 (26) | 290 (19) | 536 (30) |  |  |  |  |
| AF, n (%) |  |  |  | 0.201 |  |  |  |
| no | 2777 (87) | 1279 (86) | 1498 (87) |  |  |  |  |
| yes | 422 (13) | 209 (14) | 213 (13) |  |  |  |  |
| Hyperlipemia, n (%) |  |  |  | 0.075 |  |  |  |
| no | 2835 (89) | 1346 (90) | 1489 (88) |  |  |  |  |
| yes | 364 (11) | 142 (10) | 222 (12) |  |  |  |  |
| Smoking, n (%) |  |  |  | 0.904 |  |  |  |
| no | 2004 (63) | 930 (62) | 1074 (63) |  |  |  |  |
| yes | 1195 (37) | 558 (38) | 637 (37) |  |  |  |  |
| Drinking, n (%) |  |  |  | 0.154 |  |  |  |
| no | 2490 (78) | 1141 (77) | 1349 (79) |  |  |  |  |
| yes | 709 (22) | 347 (23) | 362 (21) |  |  |  |  |
| CHD, coronary heart disease; AF, atrial fibrillation.  P value is for the test of difference between the inclusion and exclusion group. | | | | |  |  |  |

| **Table S2.** Demographic and clinical characteristics of patients with different stroke subtypes | | | | |
| --- | --- | --- | --- | --- |
| Variables | LAA  (n = 604) | CE  (n = 308) | SAO  (n = 576) | p |
| Age, mean (SD) | 70 (12) | 75(11) | 69 (11) | <0.001 |
| Male n (%) | 402 (67) | 167 (54) | 344 (60) | <0.001 |
| Stroke, n (%) | 188 (31) | 86 (28) | 135 (23) | 0.012 |
| Hypertension, n (%) | 492 (81) | 238 (77) | 471 (82) | 0.226 |
| Diabetes, n (%) | 239 (40) | 94 (30) | 252 (44) | <0.001 |
| CHD, n (%) | 90 (15) | 122 (40) | 78 (14) | <0.001 |
| AF, n (%) | 0 (0) | 209 (68) | 0 (0) | <0.001 |
| Hyperlipemia, n (%) | 58 (10) | 27 (9) | 57 (10) | 0.860 |
| Smoking, n (%) | 250 (41) | 97 (32) | 211 (37) | 0.012 |
| Drinking, n (%) | 155 (26) | 60 (20) | 132 (23) | 0.174 |
| SDP, mean (SD) | 153 (22) | 151 (23) | 156 (21) | 0.002 |
| DBP, mean (SD) | 87 (14) | 86 (16) | 89 (14) | 0.021 |
| NIHSS, median (IQR) | 3 (1,6) | 4 (1,12) | 3 (1,6) | <0.001 |
| Severity level, n (%) |  |  |  | <0.001 |
| Minor | 414 (69) | 177 (57) | 429 (74) |  |
| Mild | 104 (17) | 48 (16) | 119 (21) |  |
| Moderate | 42 (7) | 30 (10) | 22 (4) |  |
| Severe | 44 (7) | 53 (17) | 6 (1) |  |
| FBG, mean (SD) | 7.3 (3.5) | 6.9 (2.8) | 7.3 (3.3) | 0.176 |
| TG, mean (SD) | 1.6 (1.0) | 1.4 (0.8) | 1.7 (1.1) | <0.001 |
| TC, mean (SD) | 4.8 (1.3) | 4.5 (1.0) | 4.9 (1.1) | <0.001 |
| LDL, mean (SD) | 3.1 (1.1) | 2.9 (0.8) | 3.1 (0.9) | <0.001 |
| HDL, mean (SD) | 1.1 (0.3) | 1.1 (0.3) | 1.1 (0.3) | 0.530 |
| PLT, mean (SD) | 227 (75) | 206 (70) | 214 (56) | <0.001 |
| Fibrinogen, mean (SD) | 3.5 (1.1) | 3.5 (1.1) | 3.2 (0.9) | <0.001 |
| Bilateral infarctions, n (%) | 103 (17) | 129 (42) | 67 (12) | <0.001 |
| Multiple infarctions, n (%) | 459 (76) | 248 (81) | 62 (11) | <0.001 |
| DCI, n (%) | 254 (42) | 149 (48) | 2 (1) | <0.001 |
| Season, n (%) |  |  |  | 0.015 |
| Spring | 156 (26) | 71 (23) | 160 (28) |  |
| Summer | 149 (25) | 100 (32) | 176 (41) |  |
| Autumn | 140 (23) | 78 (25) | 113 (20) |  |
| Winter | 159 (26) | 59 (19) | 127 (22) |  |
| CHD, coronary heart disease; AF, atrial fibrillation; SBP, systolic blood pressure; DBP, diastolic blood pressure; FBG, fasting blood glucose; TG, triglyceride; TC, total cholesterol; LDL, low-density lipoprotein cholesterol; HDL, high-density lipoprotein cholesterol; PLT, blood platelet; DCI, double-circulation infarction.  P value is for the test of difference among the three stroke subtypes. | | | | |
